# Video Capsule Retention in Inflammatory Bowel Disease: An Unusual Presentation and Discussion of Retrieval Methods

**DOI:** 10.1155/2013/607142

**Published:** 2013-03-03

**Authors:** R. Goel, J. Hardman, M. Gulati, J. O'Donohue

**Affiliations:** ^1^Department of Gastroenterology & General Medicine, University Hospital Lewisham, Lewisham High Street, London SE13 6LH, UK; ^2^Department of Radiology, Queen Elizabeth Hospital, Stadium Road, Woolwich, London SE18 4QH, UK

## Abstract

Inflammatory Bowel Disease (IBD) is characterized by chronic inflammation in the gastrointestinal (GI) tract. Video capsule endoscopy (VCE) is widely used to investigate the small bowel, and capsule retention is the most serious potential complication. Endoscopic and surgical management has been reported, but in the absence of bowel obstruction, there is little consensus as to which should be employed. In this case report, we describe a patient who was investigated with VCE for weight loss and anaemia. He had previously undergone colectomy with ileoanal pouch formation for ulcerative colitis (UC). Capsule retention occurred at an ileal stricture and he was subsequently diagnosed with Crohn's disease (CD). We describe his medical management and successful capsule retrieval using endoscopic methods. This case also highlights the importance of screening for intestinal strictures in an atypical presentation of UC following colectomy.

## 1. Introduction

IBD is a chronic inflammatory condition comprising UC and CD. The incidence of UC varies greatly between 0.5 and 24.5/100,000 persons, while that of CD varies between 0.1 and 16/100,000 persons worldwide [1]. The precise aetiology remains unknown but is thought to be a combination of genetic, immunologic, and environmental factors. The diagnosis is made on the clinical history, laboratory investigations, and endoscopic and histologic appearances. UC is characterized by confluent mucosal inflammation which is confined to the colon. In contrast, CD is characterized by patchy transmural inflammation which can affect any part of the GI tract, though it commonly involves the ileocaecal region. Enteric fistulas and stricturing are pathological features unique to CD which distinguish it from UC. 

Video capsule endoscopy (VCE) is used for the investigation of small bowel lesions. Its main indication is for investigation of anaemia due to occult GI bleeding. Following fasting, the patient swallows a small capsule (consisting of a camera, a light source, and a wireless circuit for the transmission of signals). As the capsule moves through the GI tract, images are transmitted to an external data recorder. These data are transferred to a computer for interpretation. The capsule is then passed in the patient's stool [2]. 

We report a case of VCE retention and recovery in a patient with IBD without resorting to surgical intervention. 

## 2. Case Report

A 36-year-old man presented to the Gastroenterology Outpatients Department with a four-month history of weight loss and iron-deficiency anaemia. He denied abdominal pain and described no change in bowel habit or rectal bleeding. He had previously been diagnosed with UC approximately 20 years ago and had undergone a colectomy with ileostomy two years later. 15 years ago, he had an ileoanal pouch formed. 

**Figure 1:**
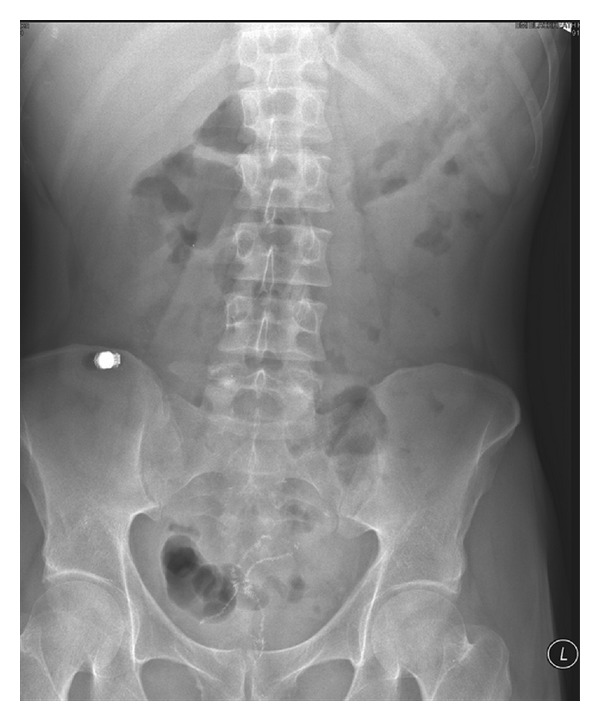
Plain abdominal X-ray showing retained capsule.

**Figure 2:**
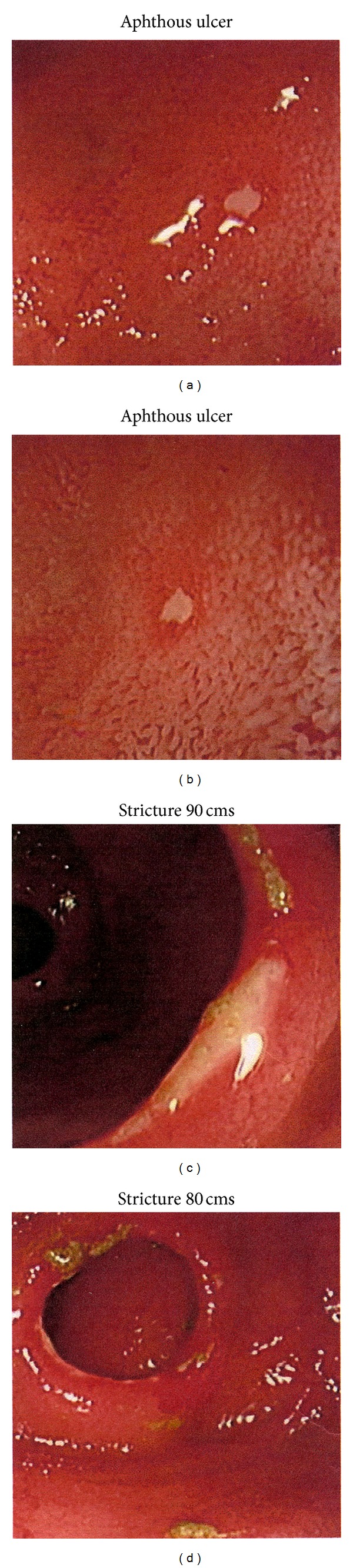
Pouchoscopy/ileoscopy showing aphthous ulcer and nontraversable stricture proximal to ileoanal reservoir.

Oesophagoduodenogastroscopy (OGD) and flexible pouchoscopy were unremarkable. VCE was performed; however the capsule was not passed (Figure 1). Pouchoscopy/retrograde ileoscopy with a paediatric colonoscope showed a normal pouch with scattered aphthous ulcers in the ileum (Figure 2). Two strictures were identified, the more significant 100 cm proximal to the pouch, which could not be traversed by the scope. Small bowel barium follow-through confirmed the endoscopy findings and showed the capsule was impacted but freely mobile proximal to this stricture. 

The capsule was retained proximal to an ileal stricture. The findings of small bowel ulceration led to a diagnosis of small bowel CD being made. There was no history of nonsteroidal anti-inflammatory drug (NSAID) consumption, vasculitis, or connective tissue disease. 

A course of budesonide was given to resolve the inflammatory component of the stricture and the patient was followed up for two months with serial abdominal radiographs, but the capsule did not pass. At repeat ileoscopy, the dominant ileal stricture could not be traversed by a paediatric colonoscope even after gentle balloon dilatation. An attempt to insert a biodegradable stent was unsuccessful. 

**Figure 3:**
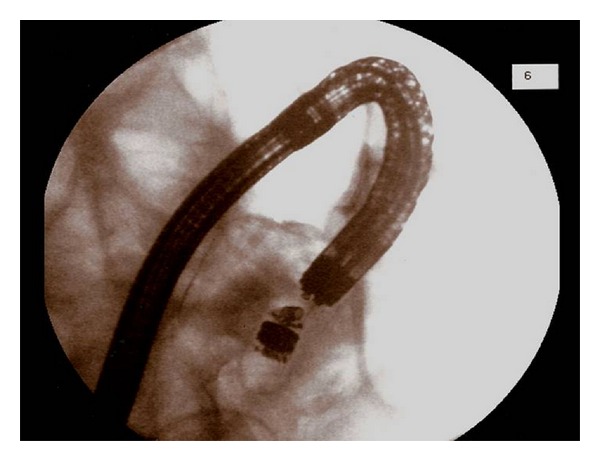
Capsule retrieval under fluoroscopy.

**Figure 4:**
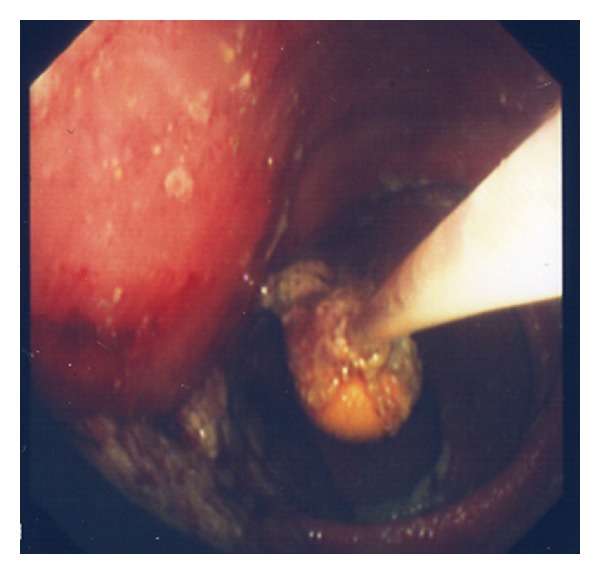
Endoscopic retrieval with a Roth net.

Azathioprine and a tapering course of prednisolone were commenced but unfortunately the capsule was still retained. After eight weeks of immunosuppression therapy, colonoscopy showed that the ileal ulceration and inflammation had markedly improved. The dominant stricture was gently dilated to 12 mm using a controlled radial expansion balloon under fluoroscopy (Figure 3), allowing passage of the scope passage and retrieval of the capsule with a Roth net (Figure 4). 

## 3. Discussion

Since acquiring a US Food and Drug Administration licence in 2001, VCE has been widely adopted for the investigation of small bowel pathology. Obscure gastrointestinal bleeding and iron-deficiency anaemia are the chief indications [2], and it has a recognised role in the investigation of CD, hereditary polyposis, and small bowel tumours [3]. VCE is generally considered to be a safe procedure and serious complications are rare. Capsule retention has been reported as occurring in approximately 0.75% of capsule examinations [4]. Small bowel strictures may be present in CD and the risk of capsule retention in these cases has been reported to be as high as 13% [5], whereas the risk of capsule retention in cases of obscure gastrointestinal bleeding has been reported to be low [6]. 

Current guidelines suggest performing initial investigations (such as a contrast study) to identify strictures where CD is a known or suspected diagnosis. This is particularly relevant for patients with obstructive symptoms or where pain is a significant feature [2, 3, 7]. The European Society of Gastrointestinal Endoscopy has specifically extended this recommendation to include patients with an atypical presentation of UC following colectomy [2]. In the present case, the patient did not describe abdominal pain or obstructive symptoms. However, given his history of colectomy, cases such as his should be considered for a patency capsule or small bowel enteroclysis prior to VCE. 

 CT, small bowel barium follow-through and MR enterography can identify physical strictures, but do not reliably predict capsule retention [8]. CT and barium studies also involve exposure to significant levels of ionising radiation [9]. The use of a patency capsule has the benefit of not involving ionising radiation and may indicate sufficient functional capacity to safely pass a video capsule [10]. However, experience with patency capsules has been disappointing, with many patients reporting delayed or nonpassage of the patency capsule [11]. A recent study has shown that CT or MR enterography is sufficient for the investigation of most patients with small bowel disease and VCE can be used as a subsequent investigation if necessary [12]. 

Both endoscopic and surgical techniques have been used in the event of capsule retention [13]. Surgery is indicated with acute small bowel obstruction and has the benefit of removing the obstructing lesion. However, in the absence of obstructive symptoms, medical management with a prokinetic drugs can be initially attempted. Immunomodulator therapy can also be employed to treat active inflammatory strictures which may allow subsequent passage of the capsule as in this case. Distinguishing between an active inflammatory stricture and a fibrotic stricture may be difficult, although MR enterography can be beneficial. 

After retention, most VCEs pass spontaneously although a significant number needs further intervention [14]. Two recently published cases have shown that surgery to retrieve an impacted VCE does not necessarily need to be undertaken immediately. In the absence of bowel obstruction, a pragmatic approach is to “wait and see” if the VCE passes spontaneously. If not, then surgery can be performed to retrieve the VCE. The same paper also reports a case similar to ours, where an impacted VCE was removed from the jejunum of a known CD patient [15]. Ultimately, there is no consensus for management of capsule retention and the choice of retrieval technique should therefore be considered on an individual case basis.
